# Supplementary material for: Integrating genomic structural equation modeling and experimental validation to unravel the genetic basis of male genital lichen sclerosus
Source: Front Immunol. 2026 Jul 16;17:1857945. doi: 10.3389/fimmu.2026.1857945 (PMC13435250; doi:10.3389/fimmu.2026.1857945)
Supplement: Supplementary file 6 [file SupplementaryFile5.zip › result/fuma_PSYCH_factor_sCCA2_focus.chr7.chr7.97099570.98715474.pop1.pdf]

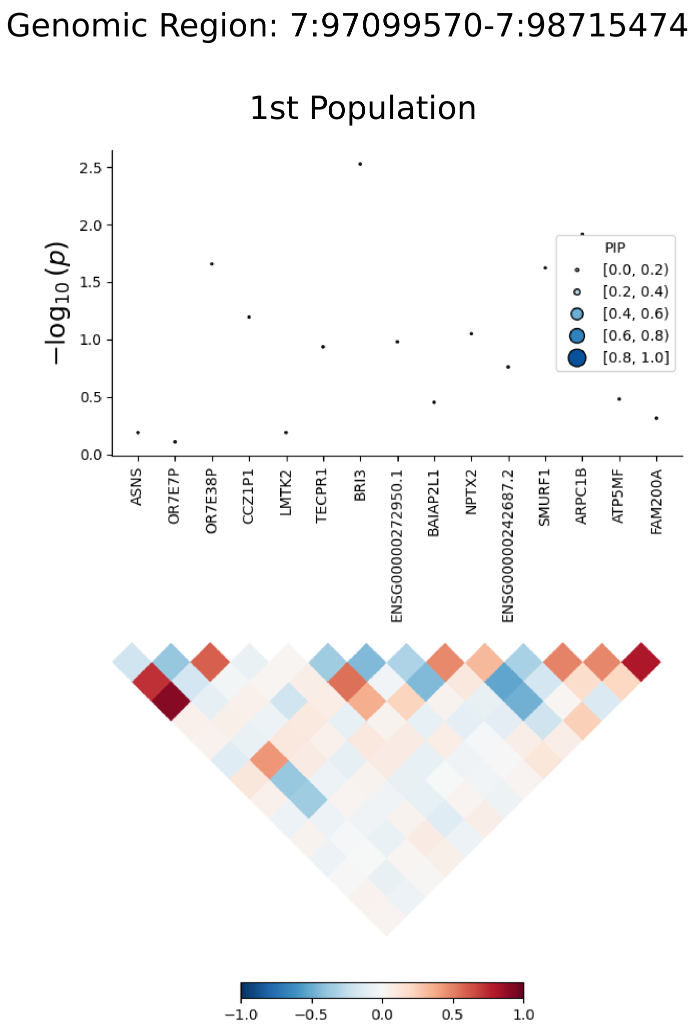
Genomic Region: 7:97099570-7:98715474
1st Population
$-\log_{10}(p)$
0.0
0.5
1.0
1.5
2.0
2.5
PIP
[0.0, 0.2)
[0.2, 0.4)
[0.4, 0.6)
[0.6, 0.8)
[0.8, 1.0]
ASNS
OR7E7P
OR7E38P
CCZ1P1
LMTK2
TECPR1
BRI3
ENSG00000272950.1
BAIAP2L1
NPTX2
ENSG00000242687.2
SMURF1
ARPC1B
ATP5MF
FAM200A
−1.0
−0.5
0.0
0.5
1.0
